# Oxcarbazepine as monotherapy of acute mania in insufficiently controlled type-1 diabetes mellitus: a case-report

**DOI:** 10.1186/1744-859X-6-25

**Published:** 2007-10-08

**Authors:** Panagiotis Oulis, Evangelos Karapoulios, Anastasios V Kouzoupis, Vasilios G Masdrakis, Konstantinos A Kontoangelos, Konstantinos Makrilakis, Nikolaos A Karakatsanis, Charalambos Papageorgiou, Nikolaos Katsilambros, Constantin R Soldatos

**Affiliations:** 1 University of Athens, Medical School, Department of Psychiatry, Eginition Hospital, Athens, Greece; 2 University of Athens, Medical School, 1st Department of Propaedeutic Medicine & Diabetologic Center, Laiko General Hospital, Athens, Greece

## Abstract

**Background:**

Type-1 diabetes mellitus (DM) is a lifelong serious condition which often renders the application of standard treatment options for patients' comorbid conditions, such as bipolar disorder I, risky – especially for acute manic episodes. We present such a case whereby the application of standard anti-manic treatments would have jeopardized a patient whose physical condition was already compromised by DM.

**Methods:**

We report the case of a 55-year-old female with a history of type-1 DM since the age of 11, and severe ocular and renal vascular complications thereof. While on the waiting list for pancreatic islet cell transplantation, she developed a manic episode that proved recalcitrant to a treatment with gabapentin, lorazepam and quetiapine. Moreover, her mental state affected adversely her already compromised glycemic control, requiring her psychiatric hospitalization. Her psychotropic medication was almost discontinued and replaced by oxcarbazepine (OXC) up to 1800 mg/day for 10 days.

**Results:**

The patient's mental state improved steadily and on discharge, 3 weeks later, she showed an impressive improvement rate of over 70% on the YMRS. Moreover, she remains normothymic 6 months after discharge, with OXC at 1200 mg/day.

**Conclusion:**

Standard prescribing guidelines for acute mania recommend a combination of an antipsychotic with lithium or, alternatively, a combination of an antipsychotic with valproate or carbamazepine. However, in our case, administration of lithium was at least relatively contra-indicated because of patient's already compromised renal function. Furthermore, antipsychotics increase glucose levels and thus were also relatively contra-indicated. Moreover, the imminent post-transpantation immunosupressant treatment with immuno-modulating medicines also contra-indicated both valproate and carbamazepine. Despite the severe methodological limitations of case reports in general, the present one suggests that OXC as monotherapy might be both safe and efficacious in the treatment of acute mania in patients with early-onset type-1 DM, whose already compromised physical condition constitutes an absolute or relative contra-indication for the administration of standard treatments, though there are no, as yet, randomized clinical trials attesting to its efficacy unambiguously.

## Background

Type-1 diabetes mellitus (DM) is a lifelong condition of glycemic metabolism, with devastating and life-threatening systemic complications. Furthermore, the overall lifelong management of type-1 DM raises crucial issues of patients' compliance to strict dietary and pharmacological regimens as well as their capacity for resilience in the face of chronic psychosocial stressors. Moreover, treatment options of patients' comorbid conditions are often restricted, as their side effects frequently threaten the already compromised physical condition of the patients.

Nowadays an abundance of literature is available attesting to both the strong relationship between bipolar disorder (BPD) and type-2 DM [1,2] and the definite or likely increased risk for the emergence of a metabolic syndrome, including type-2 DM, in newly-diagnosed patients with BPD treated with antipsychotics, especially atypical ones [3]. However, to the best of our knowledge there are no available studies or reports on safe and efficacious treatment modalities for newly-diagnosed acute mania in patients suffering from type-1 DM. In the present paper we report on such a case, treated safely and effectively with oxcarbazepine (OXC) as monotherapy.

## Case presentation

The patient was a 55-year-old married female, mother of two healthy grown-up children, with a history of type-1 DM since the age of 11. For her diabetes, she was initially treated with insulin (Novolente) once and then twice daily for 35 years. Subsequently, she was administered an intensified insulin regimen comprising insulin (NPH) twice daily and rapid-acting regular insulin three times daily pre-prandially, followed by a regimen of NPH and very-rapid acting insulin analogue (lispro) pre-prandially. During the last 3 years the patient has been treated with continuous subcutaneous insulin infusion through a pump, requiring 18–20 units/day as a basal regimen and a total of around 18–20 units/day as bolus injections. However, during the last year, due to the patient's poor compliance with the antidiabetic regimen and the required dietary pattern, her glucemic control deteriorated markedly, with frequent hypoglycemic episodes (and even comas) 2–3 times a week, followed by hyperglycemia events. In fact, the patient often felt hungry and, after the extra meals and fearing that her glucose level had risen excessively, she self-administered more insulin than required, thus causing the hypoglycemic episodes. Over the years she had also developed severe diabetic retinopathy, requiring laser treatments, as well as renal vascular complications of DM.

As a last resort to reverse this threatening situation her treating physician decided to proceed to pancreatic islet cell transplantation. Islet transplantation has been shown to normalize metabolic control in a way that has been virtually impossible to achieve with exogenous insulin [4]. A thorough clinical and laboratory pre-transplantation work-up was then performed, including CT and MRI brain scans, which yielded minor findings for microvascular brain disease. However, approximately 2 months later, the patient underwent a significant change of mental state with psychomotor restlessness, logorrhea and decreased need for sleep. A thorough clinical and laboratory work-up (including EEG and CT brain scan) was normal. Alerted by the patient's markedly and abruptly changed behavior and preoccupied by its impact on her already aggravated glycemic control, her physician referred her to a consultant in liaison psychiatry, who prescribed gabapentin up to 2 g/day and lorazepam 7.5 mg/day, without adequate clinical response. Three weeks later, quetiapine, up to 300 mg/day, was added to the patient's regimen. She remained under this mixed anti-manic treatment for 2 months, again without satisfactory clinical response. Moreover, the patient's persistently refractory mental state adversely affected her already compromised glycemic control, requiring her psychiatric hospitalization. She was highly reticent to this proposal, accepting it only reluctantly on the advice of her treating physician.

On admission, the patient was excessively talkative and exhibited pressured speech, flight of ideas, inflated self-esteem, sexual disinhibition, psychomotor excitement and increased energy with frenzied activity. Of note, no signs of cognitive impairment as assessed by the Mini Mental State Examination test [5] were detectable (score on MMSE: 29). The patient was described as an always lively person of hyperthymic temperament. However, no discrete periods of elevated or depressed mood could be traced in her history and she had never received mood-stabilizing or other psychotropic agents previously. She was diagnosed as having a manic episode according to DSM-IV-TR diagnostic criteria [6]. On the Young Mania Rating Scale (YMRS) [7] the patient initially scored 52. Her previous psychotropic medication was discontinued within the first week, with the exception of small doses of lorazepam, 1.5 mg/day, and replaced by OXC progressively titrated to 1800 mg/day for 10 days. Nausea and sedation were the only transitory side effects of OXC.

The patient's mental state under OXC improved steadily. Initially, her psychomotor excitement subsided and her sleep was normalized, followed by the submergence of her remaining symptoms and signs. Three weeks later, on discharge, her score on the YMRS had dropped to 15, showing an impressive improvement rate of almost 71.2 %. In her weekly outpatient follow-up the patient remains normothymic 8 months after discharge, again without any signs of deterioration in her cognitive functioning. We have already decreased OXC dosage progressively to 1200 mg/day, and plan its long-term continuation as a mood stabilizing treatment. Of note, the amelioration of her mental state was accompanied by a stabilization of her glycemic control, without any further hypoglycemic episodes. Likewise, regular laboratory investigations did not reveal any hyponatremia, a rare though severe possible side effect of OXC.

## Discussion

According to both the American Psychiatric Association [8] and the Maudsley prescribing guidelines [9], the standard treatment of acute mania for patients with BPD consists of a combination of an antipsychotic with lithium salts or alternatively in a combination of an antipsychotic with valproate or carbamazepine, possibly with a benzodiazepine as an adjunctive medication. However, in the case of our patient, administration of lithium salts was relatively contra-indicated as an anti-manic agent and clearly contra-indicated as a long-term mood stabilizer because of their well known nephrotoxicity and our patient's already mildly compromised renal function as a diabetic complication. Furthermore, antipsychotics – not only the atypical ones but even the respectively more safe typical or classical antipsychotics such as haloperidol – increase glucose level and were equally relatively contra-indicated in our case (see, for example, [10]), with the possible exception of ziprasidone, or aripiprazole [11]. Moreover, the imminent post-transplantation immunosupressant treatment of our patient with immuno-modulating medicines also rendered the administration of both valproate and carbamazepine (CBZ) as contra-indicated because of the attendant increased risk for leucopenia or even agranulocytosis. Besides, as has been shown in long-term follow-up studies, islet transplants' function decreases over time and thus, in order to maintain the patients' insulin independence, repeated islet transplants would have to be administered [4]. We submit that the anti-manic treatment of our patient before her hospitalization was far from adequate as gabapentin has not been proved effective, especially in monotherapy. Moreover, the dosage of quetiapine was clearly of the lowest limit of its recommended range.

Furthermore, one cannot exclude the possibility that patient's manic episode subsided spontaneously after an almost 3 month course, though we consider this hypothesis as rather implausible, given the unlikely event of the mere temporal coincidence of her recovery with the intensive inpatient treatment she underwent under OXC at a dosage of 1800 mg/day. Moreover, one cannot exclude the possibility that patient's loss of glycaemic control during the year preceding her manic episode could have contributed to its development. However, thereafter her manic state clearly contributed to the further worsening of her glycaemic control, through the loss of insight into the severity of her physical condition. Likewise, the subsequent normalization of her mood contributed to the recovery of her insight and the restoration of her compliance to the anti-diabetic regimen. Finally, we could also face the possibility that her manic episode could be the clinical manifestation of an underlying microvascular brain disease, as a complication of her 45 years of DM. However, her unimpaired cognitive function, assessed regularly over a 9-month period, jointly with the negative findings from the CT and MRI scans, provides evidence against the diagnostic hypothesis of secondary mania due to a general medical condition.

The clinical efficacy of OXC, the 10-keto analogue of CBZ, in the treatment of acute mania and hypomania is reasonably well documented in adults, but not in children and adolescents [12-14], although there are as yet no large randomized clinical trials attesting to its efficacy as a mood-stabilizer [15,16]. However, extant studies suggest that OXC could be effective as monotherapy or as adjunctive therapy in almost 60% of patients with BPD [17-19].

OXC's mechanisms of therapeutic action, similarly to those hypothesized for CBZ, could include its anticonvulsant properties and more precisely its enhancement of GABA-ergic neurotransmission through its blocking action on sodium and/or potassium channels [20], although it seems highly doubtful that their mode of anti-manic or more generally anti-bipolar action coincides with their mode of anti-epileptic action. OXC is much safer than CBZ, lacking the severe, though rare, hematologic or hepatic adverse side effects of the latter. Rash, somnolence, dizziness, headache, nausea, vomiting, fatigue and usually asymptomatic hyponatremia are common, though benign with the exception of the latter, side effects of OXC [21].

## Conclusion

Despite the obvious severe limitations inherent to case reports in general, the present work suggests that OXC as monotherapy might be both safe and effective in the treatment of acute mania in patients with early-onset type-1 DM, whose already compromised physical condition constitutes an absolute or relative contra-indication to the administration of standard treatments.

## Competing interests

The authors have received support from various pharmaceutical companies in order to attend psychiatric or medical congresses. However not from the manufacturer of oxcarbazepine, Novartis,

## Authors' contributions

PO made substantial contributions to conception and design of the present study, and in the acquisition, analysis and interpretation of the data. He was also involved in drafting the manuscript and revising it critically for intellectual content. He gave final approval for the manuscript to be published. EK made substantial contributions to conception and design of the present study, and in the acquisition of data. AK made substantial contributions in the analysis and interpretation of the data. He was also involved in drafting the manuscript and revising it critically for intellectual content. VM made substantial contributions to conception and design of the present study, and in the acquisition, analysis and interpretation of the data. He was also involved in drafting the manuscript and revising it critically for intellectual content. He gave final approval for the manuscript to be published. KK made substantial contributions to conception and design of the present study, and in the acquisition of data. KM made substantial contributions in drafting the manuscript and revising it critically for intellectual content. NAK made substantial contributions to conception and design of the present study, and in the acquisition of data. CP and NK gave final approval for the manuscript to be published. CS made substantial contributions in drafting the manuscript and revising it critically for intellectual content. He gave final approval for the manuscript to be published. All authors read and approved the final manuscript.
